# Development and Validation of the Vending Evaluation for Nutrient-Density (VEND)ing Audit

**DOI:** 10.3390/ijerph16030514

**Published:** 2019-02-12

**Authors:** Tanya M. Horacek, Elif Dede Yildirim, Melissa Matthews Schreiber, Carol Byrd-Bredbenner, Sarah Colby, Adrienne A. White, Karla P. Shelnutt, Melissa D. Olfert, Anne E. Mathews, Kristin Riggsbee, Lisa Franzen-Castle, Jesse Stabile Morrell, Kendra Kattelmann

**Affiliations:** 1 Department of Public Health Food Studies and Nutrition, Syracuse University, Syracuse, NY 13244, USA; mmatthews7290@gmail.com; 2 Human Development and Family Studies, Auburn University, Auburn, AL 36849, USA; elifdy@auburn.edu; 3 Department of Nutritional Sciences, Rutgers University, New Brunswick, NJ 08901, USA; bredbenner@sebs.rutgers.edu; 4 Department of Nutrition, University of Tennessee, Knoxville, TN 37996, USA; scolby1@utk.edu (S.C.); kolmstea@vols.utk.edu (K.R.); 5 School of Food and Agriculture, University of Maine, Orono, ME 04469-5735, USA; awhite@maine.edu; 6 Department of Family, Youth and Community Sciences, University of Florida, Gainesville, FL 32611, USA; kpagan@ufl.edu; 7 Division of Animal & Nutritional Sciences, School of Agriculture, West Virginia University, Morgantown, WV 26506, USA; Melissa.Olfert@mail.wvu.edu; 8 Food Science and Human Nutrition Department, University of Florida, Gainesville, FL 32611, USA; anne.mathews@ufl.edu; 9 Department of Nutrition and Health Sciences, University of Nebraska-Lincoln, Lincoln, NE 68588, USA; lfranzen2@unl.edu; 10 Department of Molecular, Cellular & Biomedical Sciences, University of New Hampshire, Durham, NH 03824, USA; jesse.morrell@unh.edu; 11 Health and Nutritional Sciences Department, South Dakota State University, Brookings, SD 57007, USA; kendra.kattelmann@sdstate.edu

**Keywords:** vending machines, food environment, snacks, beverages, nutrient-density score

## Abstract

*Background:* This paper describes the development and validation of the Vending Evaluation for Nutrient-Density (VEND)ing audit to comprehensively evaluate vended products based upon healthfulness, price and promotion, and machine accessibility. *Methods:* A novel vending nutrient-density score was created to determine the healthfulness of vended snack/beverage products. Field tested in United States colleges, VENDing audit (∑nutrient-density + 10 × % healthy products) and Support sub-scores (price + promotion + accessibility) were calculated for snack/beverage machines. Higher scores indicate more healthful vending options and supports for choosing healthfully. Nutrition Environment Measures Survey-Vending (NEMS-V) was used to validate the nutrient-density score for a sub-sample of machines. Sensitivity and specificity were computed by comparing the number of healthy snacks/beverages determined by NEMS-V and the VENDing nutrient-density scores. *Results:* Researchers conducted the VENDing audit on 228 snack/beverage vending machines at 9 universities within the United States and used both VENDing and NEMS-V on 33 snack and 52 beverage vending machines. Mean VENDing audit scores were 4.5 ± 2.0 (2.6, 3.4) and 2.6 ± 2.0 (0, 12) for snack/beverage machines, respectively. The number of products considered healthy assessed with both the VENDing nutrient-density scores and the NEMS-V were positively correlated for beverages (*r* = 0.687, *p* < 0.001) and snacks (*r* = 0.366, *p* < 0.05). The sensitivity was excellent for beverages (0.83) and moderate for snacks (0.69); while the specificity was moderate for both beverages (0.66) and snacks (0.50). *Conclusions:* The VENDing audit uses unique, valid, and reliable nutrient-density scoring to evaluate snacks/beverages along a continuum of healthful criteria and comprehensively evaluates the full vending environment.

## 1. Introduction

Snacking and vending products are a source of intake for Americans and others [1,2,3,4,5,6,7], contributing to overall dietary quality and potentially influencing weight management [5,8,9,10,11]. Numerous vending interventions have attempted to improve children, college student, and employee dietary intake [12,13,14,15,16,17,18]. Evaluating and tracking the healthfulness of vending machine products is necessary to assess intervention and policy efforts [12,19,20,21,22,23]. Although there are a variety of tools for evaluating the healthfulness of vended products, the inconsistency prevents comparability, and most tools are either too strict or lenient to be effective [24].

To assess snack and beverage vending products meaningfully, nutritional criteria for healthfulness must be established. Previous studies have used a diversity of healthfulness criteria, from simple to complex [24,25]. The healthfulness of vending machine products can be overestimated with lenient criteria, such as evaluating products on only one criteria (i.e., fat content). Similarly, the healthfulness of products can be underestimated with excessive criteria, such as evaluating the content of numerous macro- and micro-nutrients within products [25]. The majority of previously established healthfulness criteria were based on an all-or-nothing dichotomy (i.e., meets calorie, saturated fat, and sugar criteria, etc.), specifically to be considered a healthful product it had to meet all identified healthfulness criteria [24,25]. To be comprehensive, vended product healthfulness criteria should include a variety of nutritional parameters, however products should not be required to meet all parameters to be considered healthful [25,26,27,28,29,30,31]. It is unreasonable to expect a snack-sized vended product to meet a substantive list of nutrient parameters to be considered healthy, a more balanced approach (averaging of nutrient contributions) is imperative [25,30,31].

Few audit tools evaluate other factors that might influence what a consumer buys from a vending machine [24]. Factors beyond taste or food preferences that might influence vending purchases include pricing, product promotion, and nutrition information, but are rarely reported collectively on vending audits [24]. The price of vending machine products strongly influences consumer purchasing patterns [4,12,13,20,21,32,33] and when healthier options are offered at a reduced cost, the sales of healthier options increased [4,13,21,32,33]. Consumers of vended products are also influenced by product logos, labels, and advertisement brand marketing [25,34,35]. Given that consumers cannot view nutrition labels on a vended product prior to purchase, front of package cues might be informative. Color-coding healthier snacks with green marks and red for unhealthy snacks have been shown to increase consumer purchase of healthier snacks from vending machines [36]. Researchers have reported increased sales of healthier products with increased promotion of healthful products [13,20,32,33]. 

Machine accessibility is another important consideration when evaluating the supports for choosing healthy snack foods. In schools, where vending machines are readily accessible and turned on throughout the day, students’ dietary intake was negatively affected as they consumed sweeter vended products [1]. Unfortunately, most vended products typically provide minimal nutritional value [19,37,38,39,40,41,42,43], making it difficult for consumers to choose healthy products. When the availability of healthier products in vending machines is increased, healthier product sales consequently increase [4,13,20,44].

Most vending audit tools are focused on one or two macro-nutrients or require a snack to meet a full list of the dietary guidelines to be classified as healthy. Additionally, the diversity of tools has decreased the comparability between studies. Therefore, a new comprehensive audit tool is necessary that is easy to use, evaluating the healthfulness of snacks/beverages based upon nutrient-density and the environmental supports for making healthy choices. This research explains the development and validation of a universal Vending Evaluation for Nutrient-Density (VEND)ing audit, that evaluates snacks/beverages based upon nutrient-density to determine healthfulness along a continuum and evaluates the environmental supports for making healthy choices based upon: price, promotion and machine accessibility. This online audit system provides users with lists of nutrient-density scored snack/beverage vended products, audit training, data entry portal, and feedback with comparison to benchmark data.

## 2. Methods

### 2.1. Overview

This study had two components. Part one developed an inventory of items for the audit; conducted expert, cognitive, and pilot tests to revise the audit. VENDing was field tested at a convenience sample of college campuses in the Northeast, Midwest, and Southern United States. For part two, the developed tool (i.e., VENDing) was validated via comparison to Nutrition Environment Measures Survey-Vending (NEMS-V) [45]. Data were collected in 2016 and analyzed in 2018. Institutional Review Board approved this project as exempt given this was an environmental assessment. 

### 2.2. Methods: Instrument Development

#### Development of Inventory Items for the Audit

A comprehensive vending machine assessment tool was developed to provide a scoring mechanism to capture the overall healthfulness of vending machine environments [25]. Reviewing and comparing the current vended product assessment literature, the VENDing audit tool was developed [24,25]. The VENDing audit variables selected for inclusion were product healthfulness, price, promotion, and machine accessibility [1,4,12,13,20,24,25,32,33,34,35]. 

The nutrient-density score created to determine the healthfulness of vended products was based upon nutritional parameters set by the Institute of Medicine (IOM), daily values (DV), 2010 Dietary Guidelines for Americans, and Smart Snacks in School: USDA’s “All Foods Sold in Schools” [26,27,28]. 

All snack products were evaluated with 12 nutrient categories: calorie, saturated fat, trans-fat, sugar, sodium, fiber, calcium, iron, potassium, vitamin C, vitamin D, and vitamin E content (Table 1) [25,26,27,28]. A snack product was awarded 1 point for each healthfulness nutrient parameter met. Total nutrient-density scores could range between (0, 12) points. Given the insignificant nutrient value provided in gum and mints, they were excluded from analysis. All beverage products were categorically evaluated based on type and caloric content (Table 1) [25,26,27,28]. Beverage products were scored based on a 2-point scoring system. The nutrient-density score was used to classify vended products as healthy, somewhat healthy, and unhealthy.

To facilitate the ease of evaluating and coding vended products, the authors created a master list of scored vended products. Nutrient-density scores were computed by analyzing existing nutrient information for 228 snacks and 123 beverages vended products [37]. Reviewing the distribution of nutrient-density scores, none of the 228 snacks evaluated scored greater than “7” for nutrient-density, therefore healthfulness classification was divided into tertiles. Snacks classified as healthy scored ≥ 5 for nutrient-density. Somewhat healthy snacks scored 3 or 4 for nutrient-density. Unhealthy snacks scored ≤ 2 for nutrient-density [25]. Healthy beverages scored nutrient-density score of 2. Somewhat healthy scored 1 for nutrient-density. Unhealthy beverages scored 0 for nutrient-density [25]. 

The VENDing Audit was designed to evaluate individual vending snack or beverage machines. Qualtrics survey software (Qualtrics, Provo, UT, USA) was used to administer the VENDing Audit online. Auditors recorded the total number of snack/beverage slots, filled slots, and the number of different products. Within the Quatrics survey, snacks/beverages were categorized by their nutrient-density score. To determine a snack/beverages nutrient-density score, an auditor referred to the master snack/beverage nutrient-density list to easily identify a product by name, brand, and package weight. All snacks/beverages within a score category (0, 1, etc.) were listed alphabetically, so the number of each found snack/beverage could be entered. Should a vended snack or beverage item be missing from the master list of scored vended products, auditors can submit the product name, serving size and cost information for up to eight items on the Qualtrics survey. These new vended items are regularly used to update the master list of scored vended products.

The following variables were created for this study to assess environmental supports for choosing healthy: product price and promotion, and machine accessibility. Each question was rated on a semantic differential scale: 1 = no or more unhealthy evidence, 2 = some or balanced healthy/unhealthy evidence, 3 = primarily healthy evidence. 

*Product price* was determined by doing a snack or beverage pairing price analysis. The auditor recorded the prices of three healthful snacks (nutrient-density score ≥ 5) to three comparable (type: candy or chips, etc. and package size) unhealthy snacks (nutrient-density score ≤2) [25]. For the beverage pairing price analysis, the auditor recorded the “prices of three healthy beverages (nutrient-density score = 2) and three comparable (type: soda, milk, etc. and container size) unhealthy beverages (nutrient-density score = 0) [25]. In addition, the auditor completed a summary question regarding the average price comparison for healthy and unhealthy vended products of comparable type and size [25] the best semantic differential statement based upon their pricing analysis: healthy snacks/beverages are more expensive than comparable unhealthy options = 1; all snacks/beverages are comparably priced = 2; healthy snacks/beverages are scored less than comparable unhealthy options = 3.

*Product promotion* was the presence of nutrition information and/or logos on vending machines and products, and green eating promotion information. Consumers use a variety of different criteria beyond taste to select vending products and how products were promoted influences behavior change [14,46,47] This construct was assessed through three questions. (1) Presence of nutrition information labels on vended product packages or machine was scored: none = 1, general on machine = 2, and specific information about products in the machine = 3. Examples of general nutrition information included: no/low/reduced calories, sugar, or sodium; high/good source of vitamins or minerals; or a fruit/vegetable serving [25]. Specific nutrition information identified healthier options on the vending machine or products. (2) Vending logos were scored: Only unhealthy = 1, both healthy/unhealthy = 2, Only healthy = 3. (3) Green eating promotion highlight products as local, organic, or sustainable; Green eating promotion was scored: none = 1, general = 2, and creative/original = 3 [25]. 

*Snack accessibility* was uniquely applied for adult and children type of environments evaluated: (1) percentage of empty slots versus (2) vending machine hours of operation. For work/university environments, snack accessibility equates to the availability of snacks. Not appropriate = 1 when ≥ 50% of machine items were empty by the end of the day; Somewhat appropriate = 2 when 25–50% of machine items were empty by the end of the day; and Appropriate if = 3 when <25% of the machine items were empty by the end of the day. When used for primary /secondary school environments, snack accessibility equates to vending machine hours of operation. A machine would be scored: Not appropriate = 1 when machine was on for >50% of the school day; Somewhat appropriate = 2 when machine is on for 25–50% of the school day = 2 and Appropriate = 3 when machine was on <25% of the school day [25].

Demographic information collected for each vending machine included: type of environment (school, worksite, university, mall, etc.); building types (residence halls, academic buildings, libraries, recreation facilities, offices, and other); type of vending machine, beverages (hot/cold), snacks, mixed snacks/beverages, meals, other.

Scores were computed for snack and beverages machines according to Table 2. Higher VENDing and Support scores indicated healthier products and environments and lower VENDing and Support scores indicated unhealthier products and environments. The VENDing and Support snack and beverage scores can be used to universally compare vending machines among different environments.

### 2.3. Expert, Cognitive and Pilot Testing

The VENDing audit was cognitively tested with seven research assistants to ensure the items were interpreted accurately for a variety of vending machine types. The VENDing audit also was reviewed by five experts in food services, nutrition, and public health from various institutions to establish content validity. Cognitive testing and expert review resulted in improved wording of questions and response choices. 

The audit was pre-tested in fall 2013 at the lead author’s institution. The audit was tested on a variety of vending machine types to insure consistent interpretation between auditors and to test the effectiveness of the semantic differential scale for each question to ensure applicability across vending machine types. After refinements identified in the pretest were incorporated, VENDing was pilot-tested in spring 2014 at 11 U.S. college campuses (*n* = 206 vending machines) [25] (data not included in the manuscript). 

#### Pilot Testing Training and Interrater Reliability (IRR) 

Training and interrater reliability (IRR): student auditors completed online video-based training that taught them how to: (1) prepare for a successful audit and (2) interpret and answer each audit question with respect to the varied machine types. Then, they practiced using the VENDing audit with 2–3 different vending machines. Student auditors worked with a trained coordinator on their campus to refine their skills. Subsequently, they independently used the VENDing audit to evaluate two vending machines, which were not included in practice sessions, to establish IRR. The data were compared to the standard set to determine reliability. VENDing audits were repeated until all data collectors on a campus achieved IRR > 0.80 before they commenced with data collection. The total time for training (video, practice, and IRR) was typically three hours. IRR was satisfactory with intraclass correlation coefficient (ICC) mean 0.995 (0.829, 1.0).

Based upon pilot-tests, new vended snacks and beverages were analyzed and added to the master nutrient-density list (snacks = 285 and beverages = 138). Additionally, changes were made to the audit to improve training/IRR protocol. 

### 2.4. Audit Administration Procedures

The VENDing audit tool was used to evaluate vending machines on 9 campuses of U.S. post-secondary institutions in 7 different states during spring 2016. Each campus team independently selected vending machines for evaluation, audited them, and recorded data in the VENDing Survey. All student auditors completed online training, the IRR quiz, and practiced with the VENDing audit. 

Each campus team evaluated at least one snack and one beverage machine in each building. In general, campus teams chose the busiest buildings on campus for this VENDing audit. Vending machines with the highest traffic flow were selected for evaluation, i.e., machines on the main floor of a building. To insure a consistent/static view a vending machine for the audit, research assistants used digital cameras or smartphones to take photographs of the vending machine selected for evaluation. Photographs were taken of the front, right, and left side of each vending machine. Photographs of machine contents were also taken. The photographs allowed student auditors to capture content at one point in time avoiding differences due to consumer use. Additionally, student auditors could refer to the pictures as they completed the audit. An audit could be completed on site with appropriate internet access, but it was most effective to have picture proof of the vending machine and its contents in order to submit a high-quality VENDing audit. 

### 2.5. Validation of Vending Nutrient-Density Score

To establish concurrent validity a sub-sample of vending machines were evaluated with both VENDing and the Nutrition Environment Measures Survey-Vending (NEMS-V) tools [25,45]. NEMS-V is a reliable and previously validated vending machine assessment tool [25,45].When using the NEMS-V criteria, a snack product was considered healthy if all of the following criteria per portion as packaged were met: <200 calories, <35% calories from fat, <10% calories from saturated fat, 0% calories from trans-fat, <400 mg sodium, and <35% calories from sugar; non-flavored whole-grain pretzels; nuts and seeds were allowed as combination products as long as other nutrient standards were met and did not count against the total fat content of the product [45] According to NEMS-V, when a snack met all nutrient criteria for a serving but not the full package (vended snacks with more than one serving per container i.e., some baked products), they were coded yellow or somewhat healthy. Snacks coded as red or unhealthy according to NEMS-V meet none of the criteria. NEMS-V beverages scored as green or healthy included: plain water without flavoring, additives, or carbonation; 100% fruit juice or 100% low-sodium vegetable juice; skim or 1% milk; 8-oz servings of low-fat or nonfat chocolate or strawberry milk ≤22 grams of sugar, ≤35% of calories from total sugar. A long list of beverages (primarily artificially sweetened) qualified as yellow or somewhat healthy according to NEMS-V [48]. Red or unhealthy beverages according to NEMS-V included: regular soft drinks; sweetened tea, fruit drink (anything < 100% fruit juice); sugar-flavored water; regular sport/energy drinks; whole milk; and 2% flavored or plain milk. 

For VENDing, the healthfulness classification was divided by tertiles. For scoring classifications, healthy snacks were snacks that received a nutrient-density snack score ≥ 5. For beverages, healthy received a nutrient-density score = 2. 

### 2.6. Data Analysis 

Data were analyzed using SPSS (Version 24, 2016, IBM Corp. IBM SPSS Statistics for Windows, Version 24.0. Armonk, NY, USA). Snack and beverage mean VENDing scores were not normally distributed, thus non-parametric statistics were used to describe these data. Differences between mean nutrient-density, % healthy, VENDing scores, and Support sub-scores by machine type and building type were determined with analysis of variance (ANOVA) and post hoc Tukey-B. Chi Square was used to determine differences between support questions by machine types. Pearson’s correlations, T-tests and sensitivity and specificity were applied between a subset of individual vending machine scores evaluated by both the NEMS-V and the VENDing Nutrient-Density Score. 

Sensitivity and specificity were calculated on individual vending machines comparing VENDing Nutrient-Density Score categories to NEMS-V healthfulness categories. Sensitivity was the ability of VENDing to correctly determine healthfulness of products in comparison to NEMS-V. Sensitivity = (true positive (healthy/green))/(true positive (healthy/green) + false negative (unhealthy/green)) = probability of being determined as healthy when NEMS-V indicates healthy. Specificity was the ability of VENDing to correctly determine the unhealthy products in comparison to NEMS-V. The ability of the audits to classify vending products as unhealthy is called the test′s specificity. Specificity = (true negative (unhealthy/red))/(true negative (unhealthy/red) + false positive (healthy/red)) = probability of being determined unhealthy when NEMS-V indicates unhealthy.

## 3. Results 

Most machines audited sold only snacks (*n* = 100, 81.3%) or cold beverages (*n* = 105, 90.5%), however there were a mix of other type of machines as represented in Table 3. Machines assessed were distributed fairly equally between the Northeast, Midwest and Southern United States. The VENDing snack and beverage scores are reported in Table 4. A total of 228 vending machines were assessed on 9 university campuses. The mean snack nutrient-density score was 2.98 ± 0.63 and the percent healthy for snack machines was 15.7 ± 14.3 (0, 100). The mean beverage nutrient-density score was 0.62 ± 0.36 with the mean percent healthy for beverage machines was 20.1 ± 17.2 (0, 100). Only 12.2% of snack and 30.8% beverage machines evaluated had ≥ 25% total healthy products. There were no significant differences in mean nutrient-density, % healthy, VENDing scores or support sub-score by machine type. The only significant difference detected by building type was the snack’s mean nutrient-density: Residence halls (*n* = 26) scored significantly lower than those audited in Recreation services (*n* = 9) 2.72 ± 0.34 compared to 3.47 ± 1.15 (*p* = 0.006) (Table 5).

Although 92.2% of machines were appropriately accessible, significantly more mixed machines (beverages with snacks or prepared food) were less accessible, indicating they required restocking (Table 6). As for price comparisons, for both snack and beverage machines 58.6% of the healthy and unhealthy products were equally priced, with only 17.2% of machines scored as healthy having snacks that were less expensive than those that were unhealthy. There were no significant differences in pricing between machine type. Most machines, 79.4%, had no nutrition information, and there were no differences by machine type. As for marketing, 90.1% of snack machines had healthy or no logos, whereas, beverage machines were split 47% unhealthy logos and 53% healthy or no logos, which was significant by machine type. Most machines had no green eating promotion, however significantly more snack/beverage mixed had some general green eating promotion (Table 6). 

For the validation study, 33 snack machines and 52 beverage machines were compared with both VENDing nutrient-density score and NEMS-V. Comparisons of the healthy snack and beverage product availability evaluated by both methods are shown in Table 7. The mean number of healthy snacks using the nutrient-density score was 4.2 ± 3.6 (0, 15) (% healthy 15.1 ± 9.6) compared to 2.6 ± 3.9 (0, 16) (% healthy 7.1 ± 10.0) using the NEMS-V method. A significant weak correlation existed between the two methods regarding the mean number of healthy snacks. The mean number of unhealthy snacks determined using the VENDing nutrient-density score compared to NEMS-V were not significantly different. (Table 7). The mean number of healthy beverages using the Nutrient-Density Score was 3.6 ± 4.7 (0, 24) (% healthy 16.8 ± 13.2) compared to 2.8 ± 4.3 (0, 24) (% healthy 13.2 ± 12.5) using the NEMS-V method. A significant correlation existed between the two methods regarding the mean number of healthy beverages, and the mean number of unhealthy beverages, however, all respective means were significantly different (Table 7). Comparing VENDing Nutrient-density to NEMS-V, the sensitivity was excellent for beverages machines = 0.83 and moderate for snacks machines = 0.69; while the specificity was moderate for both beverages = 0.66 and snacks = 0.50 (Table 8).

## 4. Discussion 

This study describes the development and evaluation of the VENDing audit, a new tool to evaluate the overall healthfulness of the snack and beverage products and the vending machine environment. Higher VENDing scores indicate healthier vending products. Overall, since the average VENDing scores for snacks (4.5 out of 17) and beverages (2.6 out of 12) were relatively low, significant improvements could be made to the healthfulness of vending machine offerings. The majority of evaluated vending machines were predominantly stocked with snacks and beverages of low nutrient-density. These findings about vending machine offerings are consistent with those of other researchers who have concluded that the majority of vended products are of minimal nutritional value [19,37,40,42,49]. Additionally, only a small percentage of all machines contained general or specific nutrition information. Positively, most evaluated machines were appropriately accessible for a worksite/university environment and at least provided healthful products at comparable prices (≤unhealthful products). 

Vending consumers frequently select unhealthy snacks despite implementation of a vending policy dictating more healthy snacks [50]. Thus, simply improving healthful product availability/accessibility may be insufficient to encourage consumption of healthful vended products [50], promotion of healthful items is also necessary [14,44]. Mason and colleagues found that when unhealthy snacks were replaced by healthy snacks during the 100% Healthier Snack Vending Initiative in Chicago, 88% of vending machine patrons liked the healthier snacks that were offered; additionally, average monthly sales increased following initiation of the project [44]. To influence consumer behavior and promote a healthful vending environment, an extensive vending machine policy targeting multiple aspects (product healthfulness, pricing, and promotion) may be necessary [50]. Primary and secondary schools [22,23,51,52,53] are more pro-active than post-secondary institutions/employers in terms of setting vending nutrient/snack policies [17]; however, monitoring results convey difficulties that some schools face in actually meeting the guidelines [22,23] One study found, a combination of sufficient, price-reduced, healthy vending options with health promotional messaging increases healthy product purchases from vending machines [46], however, it is still inconclusive of the sustained effect of pricing changes or the effect of these vending interventions on profits [15,16,54].

The differences in the mean nutrient-density scores for snack machines between residence halls and recreation services, indicates more healthful options are available to gym patrons. This fact is encouraging, but also indicates improvements can be made to the machines accessible to all students on a regular basis.

The validation of the nutrient-density score of the VENDing audit with the previously validated Nutrition Environment Measures Survey-Vending (NEMS-V) tool [25,45] results are encouraging. The nutrient-density score of the VENDing audit appears to be a valid method for evaluating the healthfulness of vended product based on significant correlations and the sensitivity and specificity results between the two tested methods. Although significant correlations were present between the VENDing nutrient-density scores and the NEMS-V methods, significant differences between vended products were also evident. VENDing nutrient-density scores were comparable to NEMS-V for assessing the healthfulness of beverages but slightly less predictable for the healthfulness of snacks and for the unhealthy beverages and snacks. These differences are likely due to differences in the extensiveness for how the nutrient criteria are applied in the VENDing audit and NEMS-V. NEMS-V has strict criteria requiring a vended product to meet ALL nutrient parameters to be considered healthy [25], whereas VENDing nutrient-density builds a healthy score based upon the variety of nutrients meeting criteria. With decent sensitivity for assessing the healthfulness of beverages and snacks, the VENDing nutrient-density utilized a more practical scoring system to determine product healthfulness in which nutrient parameters are evaluated individually and progressively contribute to the overall nutrient-density score. The mean percentage of healthy vended products determined by VENDing nutrient-density and NEMS-V methods are consistent with previous vending and NEMS-V research that most vended snacks are of low nutritional value [14,26,37]. In another NEMS-V study, the percentage of healthy vended snacks ranged from 0% to 20%, and the percentage of healthy vended beverages ranged from 6% to 33% [48].

Based on the significant differences between VENDing and NEMS-V methods regarding the healthfulness of vended products, the VENDing nutrient-density scoring approach allows for a few more vended products to be classified as healthful. Differences were more obvious for snacks in part given the snack VENDing nutrient-density scoring spectrum (0–7) is larger than the nutrient-density range for beverages (0–2). Healthfulness classifications for beverages using the VENDing nutrient-density scores and the NEMS-V are more similar, contributing to the stronger correlations and less pronounced differences between the two methods. The nutrient-density scoring of the VENDing audit may allow for a more inclusive representation of vended product healthfulness [25]. 

There are several strengths associated with this study. The use of digital photography was helpful. Digital photography is advantageous in nutrition research but is fairly new to vending machine assessment studies [25,55,56,57]. Digital photography facilitates rapid data acquisition, researcher convenience, and allows for uninterrupted evaluation of the food environment [25,55,56,57]. Digital photography is a highly accurate, reliable, and time-effective way to evaluate the vending, food and nutrition environment. However, the use of digital photography is only useful for vending machines in which products are clearly visible to consumers. For vending machines that are either digital or non-transparent, vended products are not directly visible to consumers, thus a complete assessment of the machines cannot be conducted even with the assistance of digital photography. Methods are available to rate or score the healthfulness of vending products [24,42,45,58] and other aspects of the vending environment [19,24,41,59]. The VENDing audit established in this study is unique in that the healthfulness analysis is based upon product nutrient-density and comprehensively compares indicators of the vending machine environment including: availability, price, and promotion, and accessibility. Finally, the training and data collection tools and portal are all provided on a web-based server and analysis and comparative feedback are provided to audit users.

A few limitations exist. VENDing was tested on a convenience sample of college campuses. Knowing if their vending was managed by contract company or had nutrient vending policies would add value to the results, and the VENDing audit will be modified to collect this information. Although the VENDing audit is a comprehensive vending machine assessment tool, the training and data collection are just as time consuming as NEMS-V. At least for VENDing, the master-snack/beverage list (which is continually updated) enhances the ease of snack/beverage coding, whereas the NEMS-V calculator requires a full list of nutrition label information to determine a snack or beverage code. Future research should focus on automating the audit to be a user-friendly app and evaluating different environments beyond the university setting to determine generalizability. Additionally, assessing consumer perceptions of the healthfulness of vending could be compared to an objective VENDing audit as an additional form of validation [25].

## 5. Conclusions

The VENDing audit can be used to determine if any differences exist in product nutrient-density and between vending machine environments in different settings (i.e., schools, worksites, and hospitals). Using the VENDing audit to identify healthful and unhealthful aspects of the vending machine environment, interventions and policies can then be targeted at improving the overall healthful vending machine environment. 

## Figures and Tables

**Table 1:** Vending Evaluation for Nutrient-Density (VEND)ing nutrient-density healthfulness Criteria ^A^ [25].

**Snacks (Range 0–12)**	
**Nutrient**	**Healthfulness Criteria**
Calories	≤200 calories per package
Saturated Fat	≤10% DV ^B^ per package ^C^
Trans Fat	0% per package
Sugar	≤12.5 g per package ^D,E^
Sodium	≤10% DV per package
Fiber	≥10% DV per package
Calcium	≥10% DV per package
Iron	≥10% DV per package
Potassium	≥10% DV per package
Vitamin C	≥10% DV per package
Vitamin D	≥10% DV per package
Vitamin E	≥10% DV per package
**Beverages (Range 0–2)**	
**Score**	**Healthfulness Criteria**
0	—Sports drinks/life water/vitamin water (>50 calories per 8 fl. oz.) ^F^ —Sugar sweetened beverages/energy drinks/coffee drinks/lemonade/iced tea/all other beverages (>10 calories per 8 fl. oz.)
1	—Non-100% fruit or vegetable juice —Milk/flavored milk/non-dairy milk alternatives (>150 calories per 8 fl. oz.) —Sugar sweetened beverages/energy drinks/coffee drinks/lemonade/iced tea/all other beverages (≤10 calories per 8 fl. oz.)
2	—Water/flavored water —100% fruit or vegetable juice —Milk/flavored milk/non-dairy milk alternatives (≤150 calories per 8 fl. oz.)

^A^ Copyright permission provided by Melissa Matthews, November 30, 2018; ^B^ DV = Daily values; ^C^ In accordance with the *Smart Snacks in School: US Department of Agriculture’s (USDA) “All Foods Sold in School” Standards* exemptions to the saturated fat standard include reduced fat cheese (including part-skim mozzarella), nuts, seeds, nut or seed butters, products containing only dried fruit with nuts and/or seeds with no added nutritive sweeteners or fats, and seafood with no added fats. These products will automatically meet the saturated fat standard and receive 1 point for meeting the saturated fat criteria; ^D^ Modified from Institute of Medicine (IOM) criteria, to be equivalent to 25% of the recommended daily values (DV) for sugar and establishes a simple cut-off point to quickly and effectively evaluate snack products; ^E^ In accordance with the *Smart Snacks in School: USDA’s “All Foods Sold in School” Standards* exemptions to the sugar standard include dried whole fruits or vegetables, dried whole fruit or vegetable pieces, dehydrated fruits with no added nutritive sweeteners, dried whole fruits or pieces with nutritive sweeteners that are required for processing and/or palatability purposes (cranberries, tart cherries, blueberries, etc.), and products consisting of dried fruit with nuts and/or seeds with no added nutritive sweeteners or fats. These products will automatically meet the sugar standard and receive 1 point for meeting the sugar criteria; ^F^ fl.oz. = Fluid ounces

**Table 2:** VENDing scoring.

Score	Formula	Range
∑ Snack Nutrient-density	# Snacks Scored 1, +…# Snacks scored ≥7/Total # Snacks	[0, 7]
∑ Beverage Nutrient-density	# Beverages Scored 0, +…# Beverages scored 2/Total # Beverages	[0, 2]
% Healthy Snacks	# snacks scored 5 + 6 + ≥7/ total number of snacks	[0, 1]
% Healthy Beverages	# beverages scored 2/ total number of beverages	[0, 1]
Support subscore	(Product price + three Product promotion questions + Snack accessibility)/3	[1, 5]
VENDing Snack Score	∑ Snack Nutrient-density + 10 × (% Healthy Snacks)	[0, 17]
VENDing Beverage Score	∑ Beverage Nutrient-density + 10 × (% Healthy Beverages)	[0, 12]

# = Number and % = Percentage.

**Table 3:** Characteristics of machines audited.

Characteristics	Snack	Beverage
	*N* = 123	*N* = 117
Type of Machine		
Snacks	100 (81.3%)	
Prepared food	1 (0.8%)	
Prepared food and Cold beverages	2 (1.6%)	2 (1.7%)
Cold beverages		105 (90.5%)
Snacks and Cold beverages	13 (10.6%)	8 (6.9%)
Snacks/Prepared/Cold beverages	7 (5.7%)	1 (0.9%)
Machine Location		
Northeast	45 (36.6%)	33 (28.4%)
Midwest	40 (32.5%)	45 (38.8%)
South	38 (30.9%)	38 (32.8%)

**Table 4:** VENDing score results.

Scores	Snack		Beverage	
	*N* = 123		*N* = 117	
	Mean ± SD	Range	Mean ± SD	Range
∑ Nutrient-density	2.98 ± 0.63	[2.15, 5.67]	0.62 ± 0.36	[0, 2]
% Healthy	0.15 ± 0.14	[0, 1.0]	0.20 ± 0.17	[0, 1]
VENDing Score	4.55 ± 2.01	[2.26, 13.44]	2.66 ± 2.05	[0, 12]
Support Subscore	3.26 ± 0.37	[2.33, 4.00]	3.04 ± 0.50	[2, 4.33]
Product Price	1.88 ± 0.70		1.92 ± 0.64	
Product-Logos	2.80 ± 0.59		1.99 ± 0.97	
Nutrition Information	1.17± 0.51		1.31 ± 0.56	
Health Promotion	1.02 ± 0.20		1.05 ± 0.31	
Accessibility	2.89 ± 0.44		2.36 ± 0.43	

**Table 5:** Differences between VENDing scores by building type.

Scores	Building Type	Snack Machines			F	Beverage Machines			F
		*N*	Mean	SD		*N*	Mean	SD	
∑ Nutrient-density	Residential	26	2.72	±0.34 ^a^	3.779 **	28	0.51	±0.29	1.903
	Recreation Facility	9	3.47	±1.15 ^b^		16	0.76	±0.50	
	Academic	68	3.01	±0.59 ^ab^		55	0.61	±0.31	
	Library	10	3.30	±0.53 ^ab^		8	0.79	±0.53	
	Other	10	2.75	±0.70 ^a^		10	0.68	±0.29	
% Healthy	Residential	26	0.13	±0.05	1.795	28	0.15	±0.13	1.996
	Recreation Facility	9	0.26	±0.26		16	0.26	±0.23	
	Academic	68	0.15	±0.15		55	0.19	±0.14	
	Library	10	0.20	±0.10		8	0.30	±0.31	
	Other	10	0.14	±0.16		10	0.23	±0.15	
VENDing total	Residential	26	4.03	±0.79	2.322	28	2.05	±1.55	2.019
	Recreation Facility	9	6.08	±3.70		16	3.40	±2.76	
	Academic	68	4.50	±2.00		55	2.49	±1.72	
	Library	10	5.33	±1.50		8	3.87	±3.65	
	Other	10	4.11	±2.31		10	2.95	±1.72	
Support Sub-score	Residential	25	3.36	±0.32	1.379	27	3.16	±0.47	.848
	Recreation Facility	9	3.15	±0.41		15	3.16	±0.59	
	Academic	63	3.31	±0.34		52	3.06	±0.49	
	Library	9	3.11	±0.33		8	2.88	±0.50	
	Other	10	3.30	±0.25		10	2.93	±0.44	

F = F test; ** *p* < 0.01; ^ab^ Different subscripts are significantly different.

**Table 6:** Distribution of support question answers for all vending machines (*n* = 228).

Support Score Variables		Snacks (*n* = 100)	Snacks/Prepared/Beverages (*n* = 7)	Prepared Food & Beverages (*n* = 3)	Cold Beverages (*n* = 105)	Snacks & Beverages (*n* = 13)	χ^2^(df)
Accessibility							20.51(8) **
	Not Appropriately Accessible	0	0	0	1 (1%)	0	
	Somewhat Accessible	2 (2%)	3 (42.9%)	0	9 (8.7%)	2 (15.4%)	
	Appropriately Accessible	96 (98%)	4 (57.1%)	3 (100%)	94 (90.4%)	11 (84.6%)	
Price Comparison							7.20 (8)
	Healthy > expensive Unhealthy	25 (25%)	2 (33.3%)	1 (50%)	22 (21%)	4 (30.8%)	
	Healthy = Unhealthy	55 (55%)	3 (50%)	0	65 (61.9%)	9 (62.9%)	
	Healthy < expensive Unhealthy	20 (20%)	1 (16.7%)	1 (50%)	18 (17.1%)	0	
Nutrition Information on Machines							7.37 (8)
	No nutrition information	83 (83.8%)	7 (100%)	3 (100%)	78 (74.3%)	9 (75%)	
	General nutrition information	11 (11.1%)	0	0	21 (20%)	3 (25%)	
	Specific nutrition information	5 (5.1%)	0	0	6 (5.7%)	0	
Product Logos on Machines							57.86 (8) ***
	Only unhealthy product logos	8 (8%)	0	0	53 (50.5%)	3 (23.1%)	
	Both healthy and unhealthy	2 (2%)	0	0	7 (6.7%)	1 (7.7%)	
	Only healthy or no product logos	90 (90%)	7 (100%)	3 (100%)	45(42.9%)	9 (69.2%)	
Green Eating Production Promotion							14.27(4) **
	No promotion	98 (99%)	7 (100%)	3 (100%)	96 (94.1%)	10 (76.9%)	
	General promotion	1 (1%)	0	0	6 (5.9%)	3 (23.1%)	

**p < 0.01; *** *p* < 0.001.

**Table 7:** VENDing nutrient-density score and NEMS-V comparison of product healthfulness.

Product Healthfulness	Nutrient- Density Mean ± SD	NEMS-V Mean ± SD	*p*-Value	Pearson’s Correlation	*p*-Value
Snack Machines Compared (*n* = 33)					
Mean Number of Healthy Snacks ^a^	4.2 ± 3.6	2.6 ± 3.9	0.019	0.366	0.035
Mean Number of Unhealthy Snacks ^b^	16.6 ± 7.6	19.9 ± 9.9	0.089	0.231	0.196
Beverage Machines Compared (*n* = 52)					
Mean Number of Healthy Beverages ^c^	3.6 ± 4.7	2.8 ± 4.3	0.005	0.867	0.000
Mean Number of Unhealthy Beverages ^d^	11.5 ± 8.9	13.2 ± 10.9	0.015	0.904	0.000

^a^ Healthy snacks are defined as snacks that have received a nutrient-density snack score > 5 and as snacks color-coded as green according to NEMS-V; ^b^ Unhealthy snacks are defined as snacks that have received a nutrient-density snack score of ≤2 and as snacks color-coded as red according to NEMS-V; ^c^ Healthy beverages are defined as beverages that have received a nutrient-density beverage score of 2 and as beverages color-coded as green according the NEMS-V; ^d^ Unhealthy beverages are defined as beverages that have received a nutrient-density beverage score of 0 and as beverages color-coded as red according to NEMS-V.

**Table 8:** Sensitivity and specificity of VENDing nutrient-density scores in comparison to NEMS-V.

VENDing Scores	NEMS-V Snacks (*n* = 33)	
Nutrient-density Snacks (*n* = 33)	22 ^a^	18 ^b^
	11 ^c^	17 ^d^
	Sensitivity = 0.69	Specificity = 0.50
	**NEMS-V Beverages (*n* = 52)**	
Nutrient-density Beverages (*n* = 52)	43 ^a^	18 ^b^
	9 ^c^	34 ^d^
	Sensitivity = 0.83	Specificity = 0.66

^a^ Healthy Positive (NEMS-V green and VENDing ≥5 for snacks and = 2 for beverages); ^b^ False Positive (NEMS-V red and VENDing ≥5 for snacks and = 2 for beverages); ^c^ False Negative (NEMS-V green and VENDing ≤2 for snacks and = 0 for beverages); ^d^ Unhealthy Negative (NEMS-V red and VENDing ≤2 for snacks and = 0 for beverages).
